# Pediatrics

**Published:** 1897-03

**Authors:** Maud Josephine Frye

**Affiliations:** Clinical instructor in diseases of children, University of Buffalo


### PEDIATRICS.

CONDUCTED BY MAUD JOSEPHINE FRYE, M. D.,
Clinical instructor in diseases of children, University of Buffalo.

### AN ADDITION TO THE THERAPEUTICS OF SUMMER DIARRHEA IN INFANTS.

O. REINOCH (*Munich Medical Wochenschr.*, 1896, xliii., 421,) made a report in H. V. Rinkes's clinic on the use of serum, for its blood-thinning qualities, in severe cases of gastroenteritis. The sterilised serum of the cow was used, though horse serum is now employed since Landois proved that the latter was perfectly harmless to the human blood corpuscle. The experiments were made on fifteen children, ranging in age from fourteen days to nine months old, all of them very serious cases, some nearly moribund. These children were all artificially fed and were suffering from acute digestive disorders, and treatment was commenced two or three days after the beginning of the disease, when they had already rapidly lost flesh. Four of the patients died, two with severe lobor pneumonia and two of severe follicular disease of the large intestine. In the rest of the children the serum treatment showed a favorable influence on the disease. The general appearance improved, the eyes became clearer, cyanosis became less, the arterial vessels of the skin became injected and digestion was rapidly improved. Nourishment consisted of thin rice water during the first twenty-four to forty-eight hours. About 10 to 26 c.cm. of serum were injected under the skin of the thorax. No reaction ever occurred at the site of the puncture. In one case an eruption similar to measles, lasting two days, appeared after two weeks, without any fever. Fever was noted in two cases after the injection, and in one case rose to 38.5° C. The rapid improvement of the digestive power and of the stools, in most cases, was partly due, without doubt, to ingredients of the serum, which vitalised metabolism. Especially does common salt exert an influence on glandular action. The fact that albumin is injected subcutaneously in the serum is probably of importance, when we remember that in these cases nourishment was withheld for a number of days. In 20 c.cm. of the serum the albumin amounted to 1.5 gm., equal to the amount of albumin contained in 50 gm. of undiluted cow's milk or 150 gm. of mother's milk. This, of course, is but a small quantity. It is, however, possible to divide a larger portion of serum into two parts and inject, and, at all events, it seems to some purpose that even small quantities of nourishment should be brought into the body where all food has been stopped by the mouth. Further researches in this direction will be made.—*Pediatrics*, December 15, 1896.

---

### A SUBSTITUTE FOR COD-LIVER OIL.

Although children take cod-liver oil much more readily than adults (*Pediatrics*, January 15, 1897), and often digest it better after they have swallowed it, yet we do occasionally encounter patients in pediatric practice with whom the oil, either pure or in emulsion, does not agree. It is of interest, therefore, to note the results of a trial made by V. Noorden, in the Frankfort City Hospital, of a substitute for this remedy. This substitute is sesame oil, known to us in our childhood days as the lubricant used by the forty thieves on their door-hinges. The oil, obtained by expression from the sesamum orientale, is of a bright golden-yellow color, odorless and tasteless, or nearly so. It was tried in several hundred cases, about two hundred of which were in children between four and fifteen years of age. In only one of these cases was it necessary to discontinue the administration of the oil because of vomiting and diarrhea caused by it. Most of the children were scrofulous, so-called, or were suffering from debility following some one of the acute infectious diseases. In all of them, with the exception of the one case in which it was found necessary to discontinue the oil, marked and rapid improvement followed its administration. The usual dose was two or three tea- or tablespoonfuls a day, according to the age, and not infrequently double this amount was given. The cost of the oil in Germany is about 30 cents a quart.

---

### A DANGER OF BICYCLING.

Pediatrics (January 15, 1897,) calls attention to the danger of permanent heart injury from use of the bicycle. The danger is not alone to the young, but adults, when once warned, must look out for themselves. Children must be protected against themselves. In ordinary games, even if the child taxes himself beyond his strength, the strain is of short duration and the heart readily recovers itself when the task is accomplished ; but not so with bicycling, for here the strain is kept up perhaps for hours, and the injury done to the heart in a single afternoon may be beyond even the recuperative powers of youth to undo.

---

### AN EPIDEMIC OF GLANDULAR FEVER.

J. PARK WEST, of Bellaire, Ohio, (*Archives of Pediatrics*, December, 1896,) describes an epidemic of grandular fever occurring in a section of eastern Ohio in the practice of Dr. F. A. Korell. Ninety-six cases were observed, occurring in forty-three families. Records of all cases were kept, from which the following picture of this disease is drawn : a sudden and definite onset after a period of incubation ; a fever running its course in from four to seven days and terminating by crisis ; characteristic enlargement of the cervical lymphatic glands forming an elongated tumor, lying below the angle of the jaw anterior to the sternomastoid muscle, beginning always on one side and appearing later on the other ; enlargement in most cases of other lymphatic glands, notably the post-cervical, axillary and inguinal, and not infrequently the mesenteric and bronchial ; enlargement of the liver and spleen in a large proportion of cases ; prostration and rapidly developing anemia. It was clearly contagious and occurred chiefly between one and ten years of age. The possibility of an irregular form of mumps is excluded by the fact that no enlargement of the salivary glands was observed in any case, that no ordinary case of mumps appeared during the time and that over half of the patients had already had mumps or have since had them. But one death occurred, and that in a delicate child convalescing from scarlet fever.

This disease has been described by old-world observers, but Dr. West has the distinction of being the first in this country to recognise it.

---

### CONTAGIOUS IMPETIGO.

WILLIAM S. GOTTHEIL, M. D. (*Pediatrics*, October, 1896,) says this is a self-limited contagious disease of children, appearing in localised epidemics, and first described by Tilbury Fox in 1864.

Accompanied by a moderate fever and some gastric disturbance, there appear on the face and hands flat vesicles filled with transparent or cloudy serum. These dry up into characteristic golden-yellow crusts, which fall off in two or three weeks, leaving circular, reddened, non-ulcerated areas behind. Successive crops of vesicles may prolong the disease for two months or more. It is undoubtedly parasitic ; but, though Kaposi claims to have found it, the etiological factor is still unknown. The treatment consists in removal of the crusts with olive oil compresses, cleansing the skin with hot water and soap, boric acid solution, and the like, followed by the use of Lassar's paste :

| | | |
|---|---|---|
| R.—Acid. salicylic | | 30 grains. |
| Petrolati | | 1 ounce. |
| Zinci oxidi | | |
| Amyli | aa | ½ ounce. |

